# Promising Antibody Testing Strategies for Early Infant HIV Infection Diagnosis in China

**DOI:** 10.1371/journal.pone.0099935

**Published:** 2014-06-27

**Authors:** Xueli Su, Jun Yao, Yan Jiang, Jie Li, Jianfeng Han, Weidong Sun

**Affiliations:** 1 Beijing Center for Disease Control and Prevention, Beijing, People's Republic of China; 2 National HIV/HCV Reference Laboratory, National Center for AIDS/STD Control and Prevention, Chinese Center for Disease Control and Prevention, Beijing, People's Republic of China; 3 Kunming Center for Disease Control and Prevention, Kunming, People's Republic of China; University of Puerto Rico, Medical Sciences Campus, Puerto Rico

## Abstract

**Background:**

In China, 1.1% of people living with HIV were transmitted vertically, causing a heavy burden on families and society. Early infant diagnosis (EID) is critical for improving neonatal survival. The purpose of this study is to suggest improvement in antibody testing strategies with dried blood spots (DBSs) for EID in China through analysis of anti-HIV seroreversion of infants.

**Methods:**

A total of 280 infants born to HIV infected mothers in four diverse provinces of China where multiple subtypes coexist were enrolled. The status of the infants' infection was determined by HIV antibody enzyme immunoassay and Western blot analysis at ≥18 months of age or by convincing clinical and epidemiologic data for deceased infants. A total of 1028 DBSs were collected during follow-up, which were tested to obtain anti-HIV signal to cut-off ratio (S/CO) data.

**Results:**

For uninfected infants, anti-HIV S/CO decreased with age. Seropositivity percentage declined most rapidly at 6 months to 9 months of age and 98.7% children seroreverted by 12 months of age. For most infected infants, minimum S/CO values were obtained at ≤6 months of age. Antibody negative predictive value was 100% at ≥6 months of age. An S/CO increase ≥1.86 after three months follow-up can determine HIV infection. S/CO threshold of 3.17 can differentiate infected from uninfected infants for exposed kids at 9 months or older with sensitivity as 100% and specificity ≥94.2%.

**Significance:**

Suggestions obtained through studying seroreversion data of Chinese HIV-exposed infants help improve antibody strategies for HIV EID in China. The infection can be determined as early as 3 months of age and excluded as early as 6 months of age.

## Introduction

According to 2012 China AIDS Response Progress Report by Ministry of Health of the People's Republic of China [1], sexual transmission has become the main mode of HIV transmission and the epidemic is spreading from high-risk groups to the general population of China. Women accounted for 28.6% of the 780,000 people estimated to be living with HIV [1]. Thus, the health of newborns is challenged with HIV infection among the child-bearing female population. By the end of 2011, the number of reported HIV cases among pregnant women was 5313 and a total of 3380 live babies were born to HIV positive women [1]. 1.1% of people living with HIV were transmitted vertically [1], causing a heavy burden on families and society. The disease progresses fast in infants [2]–[5]. More than 80% of infected children must receive antiretroviral therapy before 6 months of age [6], [7]. Additionally, 20% to 25% of infected children acquire AIDS or die before turning two years old [8]. Neonatal antiretroviral therapy can slow down disease processes and reduce death risk significantly [9] or even achieve functional HIV cure [10]. Only early diagnosis can help access early antiretroviral therapy [11]. Therefore, early infant diagnosis (EID) is critical in improving neonatal survival and reducing the risk of further HIV transmission.

Passively acquired antibodies to HIV from mothers will not vanish until 12 months to 18 months of age. Maternal and infant self-generated IgGs cannot be distinguished from each other. Therefore, HIV infection cannot be diagnosed with conventional method of antibody detection and nucleic-acid based testing (NAT) is widely applied for EID in most developed countries. Although with poor performance, HIV serological antibody testing is simple, cheap, more available and remains as an important assay for EID in developing countries, like China, with poor medical conditions. A neonatal HIV monitoring project in Uganda confirmed that although the antibody testing sensitivity of 87.8% is less than that of the polymerase chain reaction sensitivity of 94.3%, it can reduce the cost of detection in ruling out HIV infection by 27% to 40% [12].

Although seroreversion data serve as the basis of antibody testing strategies for EID, this information is still limited in China. In the United States, many clinicians confirm the absence of HIV-1 infection with a negative HIV-1 antibody assay result at 12 months to 18 months of age [13]. In Puerto Rico, the mean time to seroreversion is 11.6 months (17.9 wk to 82 wk) [14]. In South Africa, 94.5% of 12-month-old uninfected infants are seronegative [15]. These seroreversion data help amend diagnostic strategies. In America, presumptive exclusion of HIV-1 infection can be based on one negative HIV-1 antibody test result obtained at ≥6 months of age, and definitive exclusion of HIV-1 infection is based on two negative HIV-1 antibody test results from separate specimens obtained at ≥6 months of age [13]. However, these previous studies were derived from countries where HIV-1 subtype B or C predominates. The latest study in Vietnam has revealed that only 22% of 12-month-old uninfected infants born to HIV-1 AE-infected mothers are seronegative [16]. In China, HIV-1 in subtypes A, B′, C, D, F, G and circulating recombinant forms (CRFs) 01_AE, 07_BC, and 08_BC coexist [17]–[22]. Therefore, the dynamics of neonatal HIV-1 antibodies and the mean time of seroreversion in China may also be different from countries like the United States and South Africa where only a single subtype predominates. The present study aims to propose antibody testing strategies with dried blood spots (DBSs) for EID by studying anti-HIV seroreversion in China where antibody screening and confirmation tests start at 12 and 18 months of age, respectively.

DBS technique is widely adopted for EID because of the difficulties of neonatal blood sampling. Only 70 µL to 80 µL of peripheral blood is required for DBS preparation. With non-hazardous characteristic and the convenience of short-term storage at room temperature, DBSs can be easily transported. A −20°C refrigerator is adequate for long-term storage. In addition, the antibody and nucleic acid stability of DBSs are satisfactory [23]–[25]. Thus, the DBS technique has great potential for infant HIV infection diagnosis, especially in China, where many areas have poor medical resources and sample transportation to qualified labs is a serious problem.

## Methods

### Sample Collection

As illustrated in Table 1, we collected 1028 DBSs from 280 infants born to HIV-infected mothers from Guangxi, Henan, Xinjiang, and Yunnan Provinces from January 2005 to April 2008, where HIV prevalence rates were relatively high [1]. All babies were required to be formula-fed. Infants were determined to be definitely HIV-infected if (i) both HIV antibody enzyme immunoassay and Western blot assay were positive at ≥18 months of age or (ii) when rigorous clinical and epidemiologic criteria for pediatric AIDS, in case of infants who died before 18 months, are satisfied. In our study, 24 infants with 85 DBS samples were confirmed to be infected and 256 infants with 943 DBSs were identified to be uninfected. Although no matched mothers' viral subtype information was available in this study, we obtained geographic distribution of HIV subtypes among pregnant women in our previous work [26] as follows. In Guangxi, CRF01_AE, CRF07_BC, and CRF08_BC coexist; in Henan, subtype B′ dominates; in Xinjiang, CRF07_BC predominates; and in Yunnan, B′, CRF01_AE, CRF07_BC, CRF08_BC and C coexist. These subtypes are also main forms circulating in China. Therefore, seroreversion data from these provinces are representative and valuable for HIV EID in China.

**Table 1:** Geographic distribution of infant DBSs collected.

Province	Number of infants enrolled	Number of DBSs collected
Guangxi	70	280
Henan	50	199
Xinjiang	80	240
Yunnan	80	309

### ELISA of HIV Antibody

ELISA kit of Vironostika HIV Uni-Form II plus O (BioMérieux, Netherlands) was used. First, 6-mm-sized DBS specimens were detached into coded 96-well microplate wells with a punch. To prevent inter-sample contamination, the used punch was swabbed with alcohol, burned, and then allowed to cool before processing the next DBS. Then, 200 µL of freshly prepared 0.5% Tween 20 in phosphate-buffered saline was added into each well. The microplate was covered and then incubated at 4°C for 48 h. The sample dilute (75 µL) was added into each well of the Elisa plate provided by the kit. Then, the DBS eluent was transferred into a microtiter plate using a pipette and 75 µL of the eluent was added to the corresponding well of the Elisa plate. Finally, the kit instructions were followed to perform HIV screening test. Each sample was tested thrice and signal to cut-off ratio (S/CO) data are presented as arithmetic means of the corresponding three S/CO values. External and internal quality controls were applied during the experiments. Control charts were mapped to ensure the testing quality.

### Statistical Analysis

Statistical significance was tested with t-test and accepted at the P<0.05 level. The SPSS/PC software version 6.0 (SPSS Inc., Chicago, IL) and Microsoft Excel 2010 (Microsoft Corporation, Edmond, WA) were used in all statistical analyses. Sensitivity and specificity were calculated from appropriate two-by-two tables.

### Ethical Considerations

This study was approved by the institutional review boards of both Chinese and Beijing Center for Disease Control and Prevention. Informed consent in written form was obtained from guardians on behalf of the baby participants involved in the study.

## Results

### Dynamic Change of Antibodies in Exposed Infants

**Figure 1:**
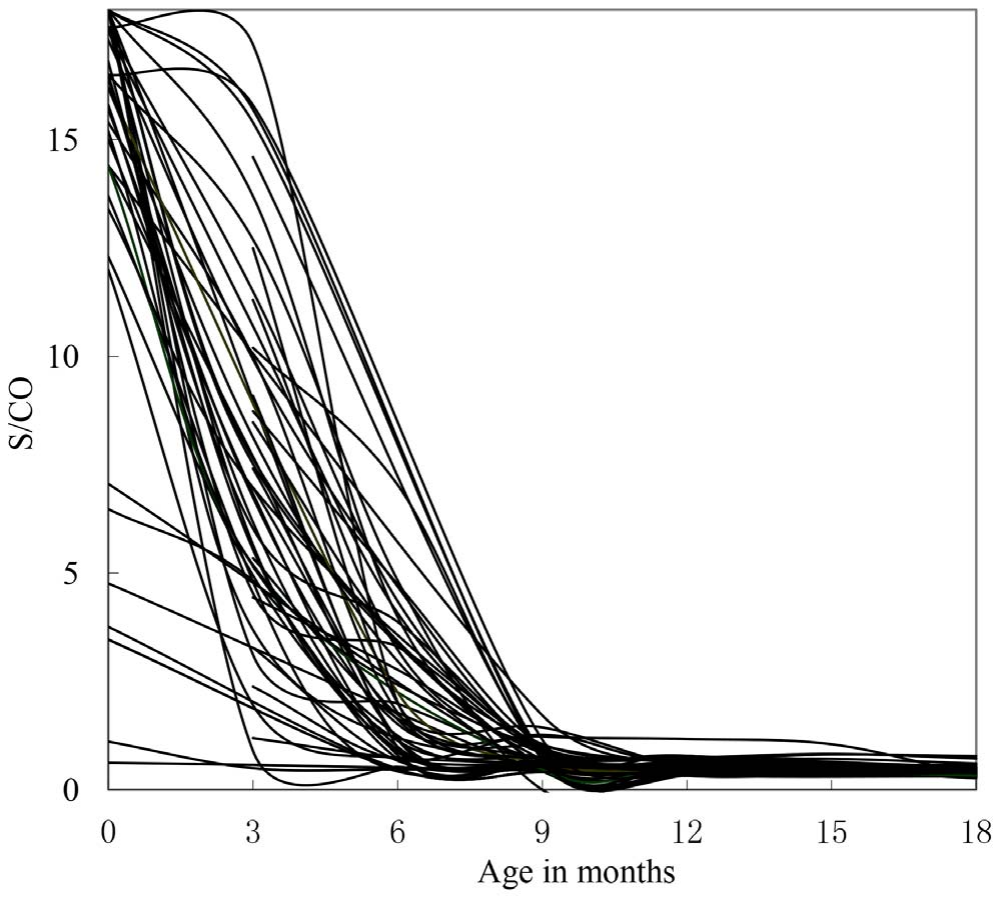
Anti-HIV S/CO values of uninfected infants at individual level. The arrow indicates S/CO = 1. The figure was derived from 312 DBSs of 56 uninfected infants whose HIV antibody S/CO values at the following months of age are available: 0 to 6, 6 to 9, 9 to 12, and 12 to 18.

**Figure 2:**
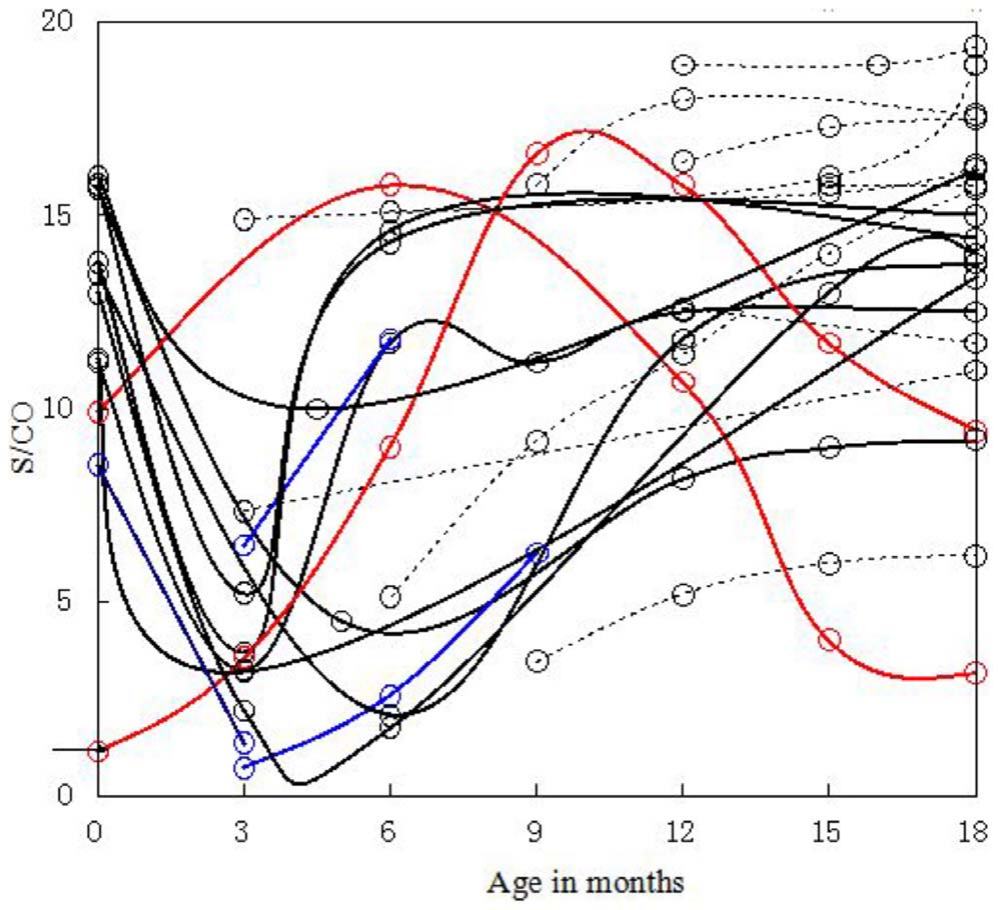
Anti-HIV S/CO values of infected infants at individual level. Black lines indicate the initial decrease and subsequent increase in S/CO; red lines indicate the initial increase and subsequent decrease in S/CO; blue lines indicate infants who died of HIV infection; and dotted lines indicate other infants. The figure was derived from 85 DBSs of 24 infected infants. The arrow indicates S/CO = 1.

Fig. 1 shows that HIV S/CO values of uninfected infants monotonically decline with month age, which is similar to the study by Moodley et al. [15], who focused on newborns in New York where HIV-1 subtype B is predominant. Contrary to those of uninfected ones, S/CO values of infected infants were much more complex. They did not decrease monotonically with increasing months of age but mainly exhibited two trends at individual levels (Fig. 2). In most cases, S/CO initially decreased and subsequently increased with minimum value occurring at ≤6 months of age.

### Dynamic Change in the Mean HIV S/CO Values

**Figure 3:**
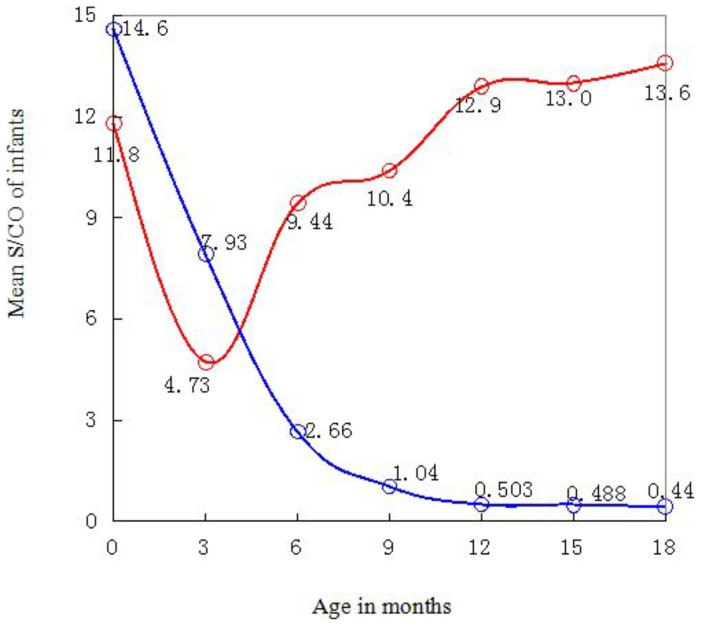
Mean anti-HIV S/CO values of infants. Red and blue lines indicate infected and uninfected infants, respectively. Calculations were based on 943 DBSs from 256 uninfected infants and 82 DBSs from 24 infected infants. At each month of age, sample sizes of infected babies were all ≥11 except at 9 months of age with 6 samples; sample sizes of uninfected babies were all ≥93.

Fig. 3 depicts the dynamics of mean HIV S/CO values of infants. For uninfected ones, mean S/CO values decreased with age, especially significantly before 6 months. But for infected babies, mean S/CO values initially decreased, reaching minimum at three months of age, and then increased.

### HIV Sero-Status of Infants

**Figure 4:**
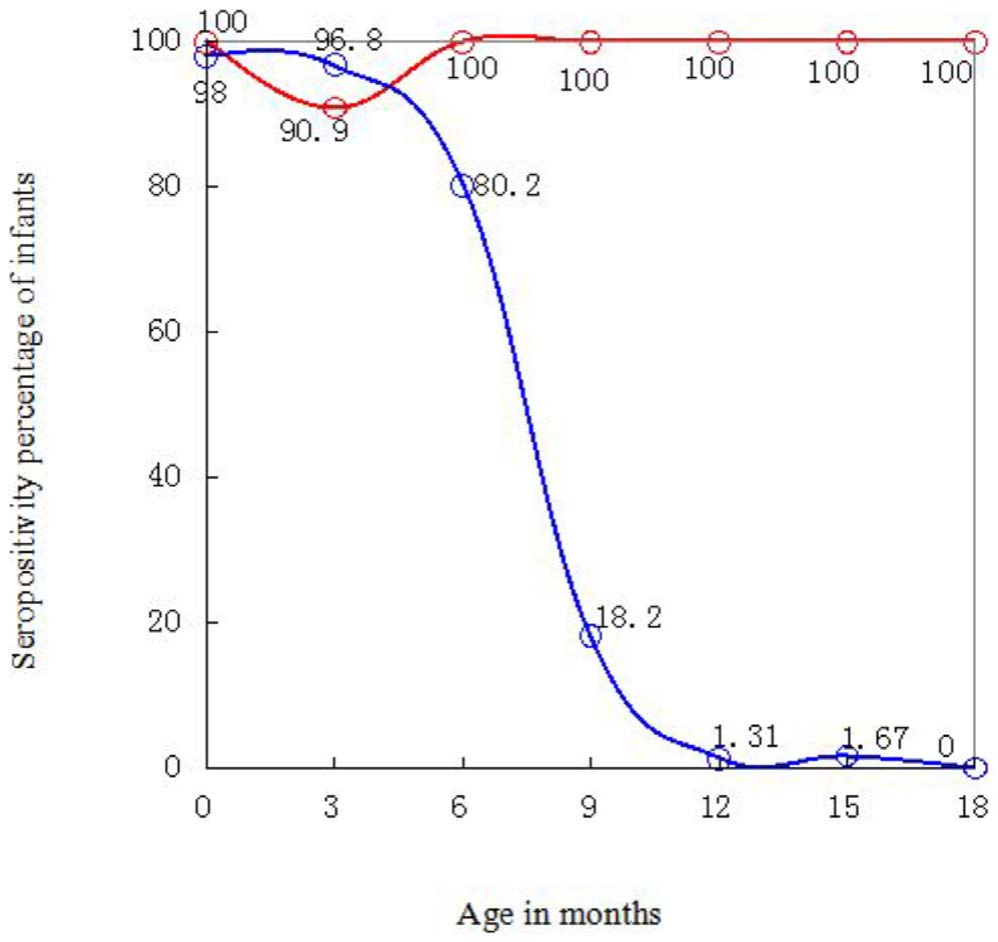
HIV sero-status of infants. Red and blue lines indicate infected and uninfected infants, respectively. Calculations were based on 943 DBSs from 256 uninfected infants and 82 DBSs from 24 infected infants. At each month of age, sample sizes of infected babies were all ≥11 except at 9 months of age with 6 samples; sample sizes of uninfected babies were all ≥93.

Fig. 4 illustrates sero-status of the infants. For uninfected ones, anti-HIV seropositivity percentage decreased with months of age and specificity increased, which is probably a consequence of exponential decay of the maternal antibodies [27]. Seropositivity percentage declined most rapidly (20.7% per month) during 6 months to 9 months of age. Only 19.8% were seronegative at 6 months of age, but as high as 81.8% at 9 months of age. Approximately 95% infants were seroreverted by approximately 10.8 months, and specificity achieved 98.7% at 12 months of age.

## Discussion

The rate of HIV-1 transmission from mother to child is 7.4% in China [1], and the calculated antibody negative predictive value was 100% at ≥6 months of age (Table 2). Thus, the negative antibody ≥6 months of age can be used to predict non-infection, which can help about 20% of uninfected infants in our study to be relived of infection. Interestingly, we found that ≥94.2% uninfected infants at 9 months or older had antibody level (S/CO) lower than 3.17 whereas those of all infected kids were higher than 3.17. S/CO threshold value of 3.17 is probably useful for China in differentiating infected from uninfected infants for exposed kids at 9 months or older. In addition, we found that during the follow-up every three months, although a monotonical decrease in S/CO at two or three serial time points did not ensure an uninfected case, increase in S/CO ≥1.86 after three months follow-up indicated infection in our study, which can help determine infection for 10 infants no elder than 9 months of age based on the samples and data available.

**Table 2:** Negative and positive predictive values with antibody ELISA testing.

Age in months	Negative predictive value	Positive predictive value
0	100	7.54
3	81.5	6.98
6	100	9.06
9	100	30.5
12	100	85.9
15	100	82.7
18	100	100

Mean S/CO value of infected infants were slightly lower than that of uninfected ones at birth, which seems to indicate effective protection of maternal antibodies, but no statistical significance was found (p = 0.058>0.05). After checking data at individual levels (Figs. 1 and 2), the protection effect of maternal antibody was found to have no effect. Instead, infant infection status cannot be inferred from HIV S/CO levels at birth, which may be because of the weakened protection from maternal antibodies in infants aroused by quick variation in the neonatal V3 loop [28].

No method is currently available to directly detect newborns' self-generated IgGs. Therefore, an indirect method was adopted as an alternative to approximate infants' self-generated antibody levels using the difference between mean S/CO values of infected and uninfected infants. The infected infants began to produce their own HIV antibodies at around 3 months to 6 months of age, which is similar to the 3 months of age proposed by Moodley et al. [15]. Antibodies acquired from mothers decayed rapidly before the infants turned 6 months old, whereas those produced by the infants themselves developed the fastest during 3 months to 6 months of age.

Special cases in this study were elaborated as follows:

- Two uninfected infants were seronegative at birth. For one mother, CD4+ T cell count, CD8+ T cell count and viral load (VL) at the third trimester (25 wk to 36 wk) were 201 cells/µL, 691 cells/µL, and <detection limit, respectively. Corresponding values at intrapartum were 216 cells/µL, 767 cells/µL and 9.21×10^3^ copies/mL, respectively. For the other mother, CD4+ T cell count, CD8+ T cell count and VL at the third trimester were 481 cells/µL, 343 cells/µL and <detection limit, respectively. Neither of the mothers' HIV-1 S/CO values at intrapartum was available. The mothers' low VL at the third trimester didn't suggest infants' seronegativity at birth because most infants whose mothers' VL during the third trimester or at production below detection limit (unpublished data) were seropositive within 48 h. Mothers' immune suppression might be one of the reasons leading to the babies' low HIV-1 antibody level and seronegativity at birth.
- S/CO values of two infected infants (illustrated with red curves in Fig. 2) initially increased, reached the maximum value at ≥6 months of age, then decreased and remained high >1 at 18 months of age. One mother's VL at production was 2.42×10^4^ copies/mL. The other mother's CD4+ T cell count, CD8+ T cell count, and VL during late pregnancy were 91 cells/µL, 2000 cells/µL, 3.42×10^4^ copies/mL. The corresponding values during intrapartum were 94 cells/µL, 1906 cells/µL, 1.43×10^4^ copies/mL. Neither mother's S/CO value was available at intrapartum. The S/CO curves of the two babies might be explained as follows: mothers' high VL led to babies' intrauterine infection and the infants had started autologous production of HIV antibodies before or since birth, which reached a high level and then decreased because of worsening immunological status.
- Three infected infants died (showed with blue color in Fig. 2). The S/CO levels of these babies were not distinguished from other infected infants. One mother's CD4+ T cell count, CD8+ T cell count and VL data at late pregnancy were 398 cells/µL, 680 cells/µL, and 6.45×10^2^ copies/mL. The corresponding values at intrapartum were 398 cells/µL, 680 cells/µL, and 2.86×10^2^ copies/mL, indicating even better immunological status and viral suppression than some mothers whose babies remained alive. Clearly, infants' deaths cannot be forecasted only on the basis of their S/CO levels or mothers' immunological and infection status.
- One infected baby's S/CO<1 at three months of age (Fig. 2), resulting in seropositive rate of infected infants at 90.9% (Fig. 4), suggests that HIV-1 infection cannot be excluded based on seronegativity for infants ≤3 months old. False seronegativity of infected newborns at particular months of age may be caused by the co-effect of maternal antibody decay and relatively slow production of the infants' own antibodies.

## Conclusions

NAT represents the gold standard for diagnostic testing of infants younger than 18 months of age and has a sensitivity of up to 90% to 100% by about 1 month of age [13]. However, for poorly equipped labs in remote areas, the antibody strategies still remains an alternative to NAT for EID.

Results of our study suggest the following HIV antibody testing strategies for EID. First, seronegativity at ≥6 months of age may exclude HIV-1 infection, similar to the strategy used in the United States for non-breastfed children younger than 18 months with no positive HIV-1 virologic test results [13]. Second, increase in the S/CO ≥1.86 after a three-month follow-up did indicate HIV infection. Third, S/CO threshold value of 3.17 can differentiate infected and uninfected infants for exposed kids at 9 months or older. With these suggestions, HIV infection can probably be determined with antibody testing as early as 3 months of age or be excluded as early as 6 months of age. Thus, antiretroviral therapy for infected infants can be performed promptly, and more young lives can be saved.

The research here has provided very valuable data for improving HIV antibody testing strategies for EID in China, but there is always room for improvement. In the future work, we'll further study in the following aspects: i) determination of the influence of specific viral subtype on the dynamic antibody curves, which could not obtained from this study because the insufficiency of matched mothers' viral subtypes; ii) evaluation of whether the proposed strategies would be affected by different diagnostic kits and different laboratories; and iii) establishment of the proper combination of antibody strategies and NAT to consider both testing availability and early diagnosis.
